# Botanical Drugs in Traditional Chinese Medicine With Wound Healing Properties

**DOI:** 10.3389/fphar.2022.885484

**Published:** 2022-05-12

**Authors:** Shuyi Ning, Jie Zang, Bingyang Zhang, Xinchi Feng, Feng Qiu

**Affiliations:** School of Chinese Materia Medica, and State Key Laboratory of Component-based Chinese Medicine, Tianjin University of Traditional Chinese Medicine, Tianjin, China

**Keywords:** wound healing, botanical drugs, multiherbal preparations, anti-inflammatory, proangiogenic, antibacterial

## Abstract

Chronic and unhealed wound is a serious public problem, which brings severe economic burdens and psychological pressure to patients. Various botanical drugs in traditional Chinese medicine have been used for the treatment of wounds since ancient time. Nowadays, multiple wound healing therapeutics derived from botanical drugs are commercially available worldwide. An increasing number of investigations have been conducted to elucidate the wound healing activities and the potential mechanisms of botanical drugs in recent years. The aim of this review is to summarize the botanical drugs in traditional Chinese medicine with wound healing properties and the underlying mechanisms of them, which can contribute to the research of wound healing and drug development. Taken together, five botanical drugs that have been developed into commercially available products, and 24 botanical drugs with excellent wound healing activities and several multiherbal preparations are reviewed in this article.

## Introduction

Recent years, chronic wounds such as venous leg ulcer, diabetic foot ulcer, and pressure ulcer have become tough issues since they are unhealed, and the course of diseases are repeated (Bowers and Franco, 2020). Nearly six million people suffer from chronic wounds worldwide (Shukla et al., 2005). Nowadays, the main treatments for chronic wounds can be divided into two main parts, surgical debridement and growth factor medicines (Powers et al., 2016). The question of surgical debridement is that it cannot cure the chronic radically. The shortcomings of growth factor medicines are the high price and side effects, which will cause more problems to patients (Knight et al., 1998; Papanas and Maltezos, 2008). Thus, development of novel wound healing therapeutics is still a research hotspot in order to provide more selections for patients with wounds.

The usage of traditional Chinese medicines for the treatment of wounds can be traced back to ancient times. According to ancient Chinese medical textbooks, various botanical drugs in traditional Chinese medicine are recommended for the treatment of wounds. As time goes on, great progress was made in demonstrating the wound healing activities of these botanical drugs and clarifying the potential mechanisms of them. Hence, in this article, we are going to make a summarization of the botanical drugs in traditional Chinese medicine with wound healing properties.

## Methods

Literatures published in English from 2000 to the present were searched in the PubMed, Web of Science, and Google Scholar databases. The search terms employed for this review included “wound healing, “ulcer,” “diabetic foot,” and “skin injure,” combined with “Chinese medicine,” “herbs,” and “medicinal plants”. Articles that included botanical drugs which were not widely distributed in China were excluded. Articles focusing on the wound healing activities of Chinese medicines which were derived from medical animals were also excluded. Meanwhile, ancient Chinese medical textbooks, including *Shen Nong’s Herbal Classic* (written by unknown authors from the Han dynasty in about 225 BC), *Compendium of Material Medica* (compiled by Li Shizhen in the Ming dynasty in 1578), and *Tang Materia Medica* (compiled by Su Jing in the Tang dynasty in 659 AD) were reviewed and botanical drugs recorded for the treatment of wounds were summarized. Among all the botanical drugs recorded, only those ones whose wound healing activities were evaluated by modern pharmacological studies were selected. Finally, five botanical drugs that have been developed into commercially available products, and 24 botanical drugs and several multiherbal preparations were reviewed in this article.

## The General Process of Wound Healing

When the skin is injured, it will trigger a well-orchestrated and complex cascade of cellular and biochemical events that aimed at repairing the damaged tissues (Wilkinson and Hardman, 2020). The wound healing process can be divided into four distinct but overlapping phases, hemostasis, inflammation, proliferation, and remodeling. After injury, hemostasis occurs immediately and blood clot will be formed serving as scaffolding for cell migration. In the inflammation phase, vascular permeability will increase, cytokines will be released, and neutrophils and macrophages will migrate to the wound site. Then, the proliferation phase will occur and this phase is characterized by the release of growth factors and cytokines, which will promote the proliferation of fibroblasts and endothelial cells. Meanwhile, collagen fibers and granulation tissue are also produced in the proliferation phase. In the remodeling phase, collagen deposition starts and the scar tissue forms. Any failure or prolongation in any phase may result in delay of healing (Janis and Harrison, 2016). In other words, botanical drugs able to accelerate any phase are potential to be wound healing agents.

## Commercially Available Products for Wound Healing Derived From Botanical Drugs

Among all the botanical drugs in traditional Chinese medicine reported to possess wound healing activities, several drugs had been extensively investigated and clinically proven to be effective, such as Centellae Herba, birch bark, calendula, aloe vera, and Curcumae longae rhizoma. In this section, those botanical drugs which have been developed into commercially available products or been clinically proven effective are summarized.

### Centellae Herba

Centellae Herba, the dry whole plant of *Centella asiatica* (L.) Urb. [Apiaceae], is a traditional Chinese medicine which grows widely in China. According to *Tang Materia Medica*, Centellae Herba was recommended to be used in the treatment of ulcers and other wounds. Nowadays, the wound healing activity of Centellae Herba has been proven in various wound models through both topical and systemic routes. Products derived from Centellae Herba, such as Madecassol^®^ and Emdecassol^®^, were already on the market for the treatment of skin problems. Several reviews were published focusing on the wound healing activity of Centellae Herba and its potential mechanisms (Brinkhaus et al., 2000; Bylka et al., 2014). Centellae Herba had been revealed to be able to promote the proliferation of fibroblast, increase the collagen synthesis, increase the intracellular fibronectin content, and inhibit the inflammation and all these attributed to its wound healing activity. Thus, readers could refer to the aforementioned reviews about the wound healing activity of Centellae Herba in animal models. In this section, we place the emphasis on the clinical trials about products derived from Centellae Herba.

In Iran, an ointment prepared with fresh leaves of *Centella asiatica* (L.) Urb. named Centiderm was tested for the efficacy against partial thickness burning in patients (Saeidinia et al., 2017). After the patients were treated with Centiderm, the burn wounds showed better healing in terms of pliability, vascularity, pigmentation *etc*, and the wounds required less days for re-epithelialization and complete healing (Saeidinia et al., 2017). Additionally, compared with silver sulfadiazine, Centiderm showed much better efficacy, indicating that Centiderm might be more suitable for the treatment of burn (Saeidinia et al., 2017). In Thailand, the efficacy of a standardized extract of Centellae Herba, named ECa 233 gel (contained madecassoside 51% and asiaticoside 38% as determined) on postlaser resurfacing wound healing was investigated in patients with atrophic acne scars (Damkerngsuntorn et al., 2020). After treated with ECa 233 gel, the wounds exhibited improved skin erythema, crusting, and wound appearance, according to the physicians’ assessment. The mechanism study showed that ECa 233 promoted the migration of keratinocyte *via* the activation of the FAK and Akt pathways, resulting in the increased expression of Rac1 and RhoA proteins, which were crucial for the formation of filopodia (Singkhorn et al., 2018). Additionally, ECa 233 could also induce cell migration *via* the activation of the ERK1/2 and p38 MAPK signaling pathways (Singkhorn et al., 2018). Due to the excellent wound healing activity of Centellae Herba, several researchers put their efforts on developing the Centellae Herba contained wound dressing. Several Centellae Herba-loaded dressings have been developed, including the hydrocolloid, electrospun double-layered nanocomposites membrane and electrospun gelatin nanofibre (Jin et al., 2015; Yao et al., 2017; Mouro et al., 2020). All these Centellae Herba-loaded dressings showed excellent wound healing activities, which indicated that Centellae Herba-loaded dressings were potential therapeutics for wounds.

### Birch Bark

Birch Bark is the cortex of various birch species, such as *Betula pendula* Roth [Betulaceae], *Betula pendula subsp. mandshurica* (Regel) Ashburner & McAll [Betulaceae], and *Betula pubescens* Ehrh. [Betulaceae]. According to *Compendium of Material Medica*, birch bark was used to treat the acute mastitis traditionally. Nowadays, birch bark is clinically proven to be effective for wound healing. Oleogel-S10 (Episalvan^®^) is a drug containing triterpene-rich extract from birch bark which had been approved for treatment of partial thickness wounds in adults in the European Union (Scheffler, 2019). Mechanism studies conducted in primary human keratinocytes revealed that birch bark triterpene extract could upregulate various mediators involved in the inflammatory phase of wound healing, resulting in a temporary inflammation (Ebeling et al., 2014). As we all know, the excessive and prolonged inflammatory phase leads to chronic wounds, whereas a temporary inflammation is necessary for wound healing (Landen et al., 2016). Meanwhile, birch bark triterpene extract could also stimulate the migration of keratinocytes and the epidermal differentiation (Ebeling et al., 2014).

Several clinical results provided the evidence that Oleogel-S10 accelerated wound healing. In the Metelmann’s study, a phase II clinical trial was conducted in patients requiring split-thickness skin graft transplantation to assess the wound healing activity of Oleogel-S10 (Metelmann et al., 2015). No adverse events were reported in the involved 24 patients, and Oleogel-S10 markedly accelerated re-epithelialization at the donor sites. In another phase III trial conducted by Frew *et al.*, the wound healing efficacy of Oleogel-S10 for the treatment of superficial partial thickness burn wounds was evaluated and compared with Octenilin^®^ intra-patient (Frew et al., 2019). Results suggested that Oleogel-S10 significantly facilitated the healing of superficial partial thickness burn wounds and it was superior to Octenilin^®^ in terms of efficacy and tolerability. Oleogel-S10 was also clinically proven to be effective for epidermolysis bullosa by phase II and III trials, and the mechanism was to promote the re-epithelialization of wounds (Schwieger-Briel et al., 2017; Kern et al., 2019; Schwieger-Briel et al., 2019).

### Calendula

Calendula [Asteraceae; *Calendula officinalis* L.] or marigold is a medical plant mainly distributed in China, Europe, India, and the United States. Various research studies proved that calendula possesses excellent wound healing activities (Givol et al., 2019). *In vitro* studies demonstrated that calendula significantly stimulated the proliferation and migration of fibroblasts, upregulated the expression of growth factors (CTGF, TGFβ1, and bFGF), and α-smooth muscle actin (α-SMA) (Fronza et al., 2009; Dinda et al., 2015; Dinda et al., 2016; Hormozi et al., 2019). *In vivo* studies found that it can accelerate re-epithelization, enhance angiogenesis, and promote collagen deposition (Preethi and Kuttan, 2009; Parente et al., 2011).

Since the mid-20th century, the Cicaderma ointment, prepared with *Calendula officinalis* L [Asteraceae], *Hypericum perforatum* L [Hypericaceae], and *Achillea millefolium* L [Asteraceae] extracts, has been marketed in Europe for the treatment of wounds, insect bites, and so on (Morin et al., 2012). Boiron Calendula cream/gel is another commercialized product in France for adjuvant treatment of irritant dermatitis and superficial burns (Pommier et al., 2004). Meanwhile, calendula was also proven to be effective for the treatment of venous leg ulcer (Buzzi et al., 2016), diabetic foot ulcer (Carvalho et al., 2016), transected tendon (Aro et al., 2015), and acute dermatitis during irradiation for breast cancer (Pommier et al., 2004).

### Aloe vera

Aloe vera [Asphodelaceae; *Aloe vera* (L.) Burm. f.] is a traditionally used medicinal plant for various skin lesions. According to *Compendium of Materia Medica*, aloe vera was used to treat exudative dermatitis in ancient China. Nowadays, it is widely used for skin problems and various types of aloe vera gels are commercially available.

Numerous research studies have proven that aloe vera possesses excellent wound healing activities with negligible toxicity *via* modulating the inflammation, increasing wound contraction, and epithelialization (Burusapat et al., 2018; Sanchez et al., 2020). Boudreau’s research found out glucomannan was a core composition of aloe vera, which stimulated the proliferation of fibroblasts and in turn improved collagen production and secretion (Boudreau and Beland, 2006).

### Curcumae Longae Rhizoma

Curcumae longae rhizoma, the dried rhizome of *Curcuma longa* L [Zingiberaceae], is a traditionally used botanical drug as a wound healer in ancient China and India. Curcumin is the main active component and has been proven to be able to arouse earlier re-epithelialization, improve neovascularization, and increase migration of various cells including dermal myofibroblasts, fibroblasts, and macrophages into the wound bed (Aggarwal et al., 2007; Akbik et al., 2014). Even though curcumin possesses powerful wound healing activities, curcumin is limited by its low bioavailability, poor solubility, and rapid metabolism (Stanic, 2017). Thus, novel formulations were explored and wound healing products derived from curcumin including CumarGOLD Gel^®^ and Psoria-Gold^®^ Curcumin Gel had been marketed in various countries.

## Botanical Drugs in Traditional Chinese Medicine With Wound Healing Activities

In addition to the aforementioned ones, numerous botanical drugs in traditional Chinese medicine have been traditionally used for the treatment of wounds, and their potential molecular mechanisms were elucidated by various studies. In this section, 24 botanical drugs (Table 1) and their wound healing activities are reviewed.

**TABLE 1:** Chinese botanical drugs and their wound healing activities.

Botanical drug and Source	Family	Extract/component/product	Experimental model	Dose and route of administration	Wound healing activity and potential mechanism	Reference
Angelicae dahuricae radix [Angelica dahurica (Hoffm.) Benth. and Hook.f. ex Franch. and Sav. or Angelica dahurica var. formosana (H.Boissieu) Yen]	Apiaceae	70% ethanol extract	Diabetic rats with full-thickness wound	1.2 g/kg once daily by oral gavage	Promoted diabetic wound closure *via* inducing angiogenesis and granulation tissue formation	Zhang et al. (2017b)
			db/db mice with full-thickness wound	1.8 g/kg once daily by oral gavage	Reduced wound area, increased in neovascularization, increased PDGF-β expression, and capillary formation	Guo et al. (2020)
			Human umbilical vein endothelial cells (HUVECs)	10–400 μg/ml	Induced cell proliferation, migration, and tube formation *via* the activation of ERK1/2, Akt, eNOS. Increased NO production and the expression of VEGF.	Zhang et al. (2017b)
			HUVECs	50–300 μg/ml	Promoted angiogenesis of HUVECs *via* activation of the HIF-1α/PDGF-β pathway	Guo et al. (2020)
Angelicae sinensis radix [Angelica sinensis (Oliv.) Diels]	Apiaceae	Extract containing 60% polysaccharide	HUVECs	11.1–100 μg/ml	Stimulated the proliferation and migration of HUVECs *via* the activation of the JNK 1/2 and p38 signal pathways	Lam et al. (2008)
		—	Zebrafish embryos	50–400 μg/ml	Increased in angiogenic phenotype in subintestinal vessels	Lam et al. (2008)
		SBD.4	db/db mice with full-thickness wound and human skin grafted on SCID mice with full-thickness wound	Topically applied SBD.4 (2 mg per wound) in 2% carboxymethyl cellulose	In db/db mice, SBD.4 stimulated wound healing with complete remodeling of the wounds. In SCID mice, SBD.4 increased granulation tissue formation and assisted in the remodeling of wounds	Zhao et al. (2006)
		SBD.4 (1%)-nanosilver hydrocolloid dressing	Chronic ulcer patients	External application	All patients’ wounds healed at the end of the treatment (day 30)	Zhao et al. (2012)
Astragali radix [*Astragalus* mongholicus Bunge]	Fabaceae	Formononetin	Mice with full-thickness wound	Formononetin (50 μM) was injected into dermis nearby wound	Accelerated wound-closure rate	Huh et al. (2011)
		—	HUVECs	0.1–50 μM	Promoted endothelial repair *via* the regulation of Egr-1 through the ERK and p38 MAPK pathways	—
		Astragaloside IV (AS-IV)	Diabetic mice with full thickness wound	10 μL AS-IV (100 mM) once daily by local delivery	Narrower wounds gaping and augmented re-epithelialization	Luo et al. (2016)
		APS2-1 (polysaccharide)	Human skin fibroblasts	1, 5, and 25 μg/ml	Promoted cell proliferation and migration	Zhao et al. (2017)
		—	Scalded mice model	Topically applied 0.5 g ointment (containing 1.55% APS2-1)	Accelerated wound healing *via* reducing inflammatory response and promoting the expression of TGF-β1, bFGF, and EGF.	Zhao et al. (2017)
Draconis sanguis [*Calamus* draco Willd.]	Arecaceae	Dracorhodin perchlorate (DP)	Rats with full-thickness wound	Topical applied DP ointment (200 μg of DP DMSO solution mixed with 16 g of Vaseline)	Reduced inflammation *via* inhibiting IL-1α and TNF-α secretion. Increased VEGF and TGF expression, and collagen deposition	Jiang et al. (2017)
			HaCaT keratinocytes	1 μg/ml and 2 μg/ml	Promoted the wound healing of HaCaT keratinocytes *via* β-catenin, ERK, p38 MAPK, and AKT pathways	Lu et al. (2021)
			HUVECs under high-glucose stimulation	7.5 μM	Promoted the angiogenesis *via* the activation of the Ras/MAPK pathway	Li et al. (2016a)
			NIH/3T3 fibroblasts	0.5–4 μg/ml	Promoted fibroblast proliferation through the activation of the ERK-CREB and PI3K/Akt/mTOR pathways	Liu et al. (2019)
Notoginseng Radix et Rhizoma [Panax notoginseng (Burkill) F.H.Chen]	Araliaceae	Notoginsenoside R1	HUVECs	10 μg/ml	Stimulated the proliferation of HUVECs and enhanced its ability of tube formation	Yang et al. (2016)
		Ginsenoside Rg1	Excision diabetic foot ulcer	150 mg/kg (i.p.)	Accelerated wound healing in diabetic ulcer through NO pathway *via* miR-23a	Cai et al. (2019)
		Notoginsenoside Ft1	HUVECs	0.25–10 μM	Stimulated angiogenesis *via* HIF-1α-mediated VEGF expression, with PI3K/AKT and Raf/MEK/ERK concurrently participating	Shen et al. (2012)
		—	Excisional wound in db/db mice	Topically applied 15 μL solution (6.7 mg/ml) once daily	Promoted the neovascularization accompanied with increased VEGF, PDGF, and FGF at either mRNA or protein levels. Reduced inflammation *via* downregulating TNF-α and IL-6 expressions	Zhang et al. (2016a)
		Ginsenoside Rb1	A rat model of second-degree burn injury	Topical application of ointment at the dose of 1.25, 2.5, or 5 g/kg	Accelerated burn wound healing *via* upregulating FGF-2/PDGF-BB/PDGFR-β gene and protein expressions	Zhang et al. (2021)
		20(S)-Protopanaxadiol	Excisional wound splinting model in db/db mice	Topically applied of 15 μL solution (0.6, 6 and 60 mg/ml) once daily	Enhanced angiogenesis *via* HIF-1α-mediated VEGF expression by the activation of p70S6K *via* PI3K/Akt/mTOR and Raf/MEK/ERK signaling pathways	Zhang et al. (2017a)
Arnebiae Radix [Arnebia euchroma (Royle ex Benth.) I.M.Johnst., Arnebia guttata Bunge or Lithospermum erythrorhizon Siebold and Zucc.]	Boraginaceae	A/S-based ointment	Dogs with full-thickness skin defect on forelimb	Topically applied a thin layer of the ointment	Promoted wound angiogenesis, collagen production, and epithelialization	Karayannopoulou et al. (2011)
		Shikonin	Human gingival fibroblasts	1 and 10 μM	Promoted fibroblast proliferation and migration *via* ERK 1/2 signaling pathway	Imai et al. (2019)
			Human keratinocytes and HDFs	1 μM	Promoted cell proliferation and showed anti-inflammatory activity *via* inhibiting the NF-κB signaling pathway	Yan et al. (2015)
Bletillae Rhizoma [Bletilla striata (Thunb.) Rchb.f.]	Orchidaceae	Polysaccharides	Mice with full-thickness wound	12.5%-crosslinked polysaccharides hydrogel topically applied	Reduced inflammatory cells, decreased TNF-α, and increased EGF secretion in polysaccharides-treated wounds	Luo et al. (2010)
			Diabetic mice with full-thickness wound	5% polysaccharides solution (50 μL) treatment once daily	Accelerated wound healing, suppressed macrophage infiltration, promoted angiogenesis *via* inhibiting the high glucose-activated NLRP3 inflammasome	Zhao et al. (2021)
Rhei Radix et Rhizoma [Rheum palmatum L., Rheum tanguticum (Maxim. ex Regel) Balf. or Rheum officinale Baill.]	Polygonaceae	Emodin	Rats with full-thickness wound	Topically applied at the dose of 100–400 μg/ml	Enhanced cutaneous wound healing *via* regulating the Smads-mediated TGF-β1 signaling pathway	Tang et al. (2007)
Rehmanniae Radix [Rehmannia glutinosa (Gaertn.) DC.]	Orobanchaceae	Aqueous extract	Diabetic foot ulcer rat model	Topically applied at the dose of 1.85 g/kg	Better developed scars and epithelialization, and improved formation of capillaries with enhanced VEGF expression	Lau et al. (2009b)
		Norviburtinal	Zebrafish embryo model	50 μg/ml	An increase in capillary sprouts formation in SIV.	Liu et al. (2011)
		Acteoside	HDFs	6.3–100 µM	The activation of proMMP-2 along with an increase in MT1-MMP expression through a PI3K signal pathway	Nan et al. (2018)
Salviae Miltiorrhizae Radix et Rhizoma [Salvia miltiorrhiza Bunge]	Lamiaceae	Nonalcoholic solution produced with a 1:3 dry herb/menstruum ratio	Rats with burn wound	Orally administered at the dose of 1 g/kg/day for 14 days	Decreased the amount of necrosis in burn wounds	Irmak et al. (2018)
		Cryptotanshinone	db/db mice with excisional wound	300 mg/kg/d by gavage for 16 days	Accelerated wound closure and increased re-epithelialization and granulation tissue formation	Song et al. (2020)
		Danshensu and salvianolic acid B	Detroit 551 human normal fibroblasts	25–200 μM and 0.1 mM	Increased cell proliferation (25–200 µM) and promoted collagen synthesis (0.1 mM)	Chen et al. (2014)
Llilii Bulbus [Lilium lancifolium Thunb., Lilium brownii var. viridulum Baker or Lilium pumilum Redouté]	Liliaceae	Steroidal glycoside 1 and 2	3T3 murine fibroblasts	5 µM	Induced production of NO and increased mRNA level of TGF-β Type I receptor, which played important roles in early wound healing	Esposito et al. (2013)
		Steroidal glycoside 1	Primary human dermal fibroblasts	5 µM	Downregulated gene expression of inflammatory, chemokine, and tissue remodeling, upregulate ECM and cell adhesion related genes to regulate basic functions of cells in wound healing	Di et al. (2020)
Glycyrrhizae Radix et Rhizoma [*Glycyrrhiza* uralensis Fisch. ex DC., *Glycyrrhiza* inflata Batalin or *Glycyrrhiza* glabra L.]	Fabaceae	Collagen sponge loaded with 72 µg soluble polysaccharide in microcapsule	Rat trauma model	Topically applied	Increased the content of hydroxyproline, promoted the proliferation of capillaries and fibroblasts, and increased number of microvessels in wound site through activating the expression of p-STAT3 and VEGF and upregulating the tanscription levels of VEGF mRNA and miRNA-21 genes	Hao et al. (2020)
		Isoliquiritin	Zebrafish skin wound model	100 or 200 μg/ml	Promoted inflammation response and facilitated angiogenesis	Liu et al. (2020)
Zingiberis Rhizoma Recens [Zingiber officinale Roscoe]	Zingiberaceae	10-shogaol	Human normal epidermal keratinocytes and dermal fibroblasts	2 and 10 µM	Enhanced the production of TGF-β, PDGF-αβ, and VEGF.	Chen et al. (2012a)
		6-dehydrogingerdione	Fibroblasts	2 and 10 µM	Upregrulated the production of growth factor, and accelerated cellular proliferation, and migration through blocking the MAPK pathway by supressing c-Jun protein levels and ERK phosphorylation	Chen et al. (2013a)
Lonicerae Japonicae Flos [*Lonicera japonica* Thunb.]	Caprifoliaceae	95% ethanol extract	Rat excision wound model	10% (w/w) extract ointment topically applied	Promoted wound healing, elevated the production of IL-10, and suppressed the production of TNF-α and IL-6	Chen et al. (2012b)
Portulacae Herba [Portulaca oleracea L.]	Portulacaceae	Fresh plant homogenate	Mouse excision wound model	A single dose of 50 mg or a twice dose of 25 mg topically applied	Stimulated wound contraction and increased the strength of wound	Rashed et al. (2003)
Hippophae rhamnoides L	Elaeagnaceae	Leaves extract	Rats with full-thickness wound	1% aqueous extract prepared in propylene glycol topically applied twice daily	Reduced wound area and increased the hydroxyproline and protein contents in wounds	Gupta et al. (2005)
			Rats with burn wound	5% extract prepared in petroleum jelly topically applied twice daily for 7 days	Faster reduction in the wound area, increased collagen synthesis, promoted angiogenesis, and increased levels of antioxidants in wounds	Upadhyay et al. (2011)
		Seed oil	Rats with burn wound	Co-administered by two routes at the dose of 2.5 ml/kg (p.o.) and 200 µL (topical) for 7 days	Increased expression of MMP-2 and 9, collagen III and VEGF in granulation tissue	Upadhyay et al. (2009)
			Sheep with 3rd degree flame burns	20 ml seed oil topically applied	Shorter complete epithelization time	Ito et al. (2014)
Urtica dioica L	Urticaceae	Crude saponins extract	Rats with full-thickness wound	20% saponins extract was prepared in vaseline and topically applied once daily	Promoted the wound healing with shorten heal time	Razika et al. (2017)
		Dried leaves extract	Rats with full-thickness wound	10% extract dissolved in glycerol topically applied on the wound at the dose of 50 µL/mm2	Accelerated wound healing with fast wound closure and improved hydroxyproline content	Zouari Bouassida et al. (2017)
Periploca forrestii Schltr	Apocynaceae	65% ethanol eluted fraction (EPFE65) derived from macroporous resin column	Rats with full-thickness wound and mouse fibroblasts	50 μg/ml for *in vitro* studies and 0.1% EPFE65 hydrogel for animal studies	Promoted wound healing *via* enhancing the re-epithelialization, promoting fibroblast proliferation, migration, and stimulating the collagen synthesis	Li et al. (2019a)
		Periplocin	Rats with full-thickness wound and mouse fibroblasts	5–20 μg/ml	Promoted wound healing *via* the activation of Src/Erk and PI3K/Akt signaling pathways	Chen et al. (2019)
Streptocaulon juventas (Lour.) Merr	Apocynaceae	Ethanol extract	Mice with full-thickness wound	Topically applied at the dose of 100 mg/kg/day	Promoted wound healing *via* inducing the fibroblast proliferation and angiogenesis, and inhibiting the inflammation	Nguyen et al. (2017)
Carthamus tinctorius L	Asteraceae	Hydroxysafflor yellow A	HUVECs and human epithelial keratinocytes	0.4, 0.8 and 1.6 mM	Enhance keratinocytes migration in a dose-dependent manner (0.4–1.6 mM). Increased the tube formation of HUVECs at 0.4 mM	Gao et al. (2018)
			Splinted excisional wound model in diabetic rats	0.2 mg locally applied once daily	Promoted wound closure and elevated the levels of VEGF and TGF-β1 in treated wounds	Gao et al. (2018)
Reynoutria japonica Houtt	Polygonaceae	20% or 40% ethanol extract or 60% acetone extract	Human gingival fibroblasts (HGFs)	50 μg/ml	Stimulated cell proliferation, migration, and collagen III synthesis	Nawrot-Hadzik et al. (2021)
Artemisia annua L	Asteraceae	Artemisia annua L.-containing nanofibers	Mouse fibroblasts	Fibroblasts were seeded onto the dressing	Encouraged the attachment, spreading, and proliferation of the fibroblast cells	Mirbehbahani et al. (2020)
Acorus calamus L	Acoraceae	80% ethanol extract of dried leaves	Rats with excision or incision wound	20% or 40% ointment topically applied once daily	Accelerated wound closure, less inflammatory cells, and higher collagen in treated wounds	Jain et al. (2010)
		70% ethanol extract of rhizome	Rats with excision wound	40 mg/kg topically applied once daily	Increased the levels of collagen, hexosamical, and uronic acid in treated wounds	Ponrasu et al. (2014)
*Triticum aestivum* L	Poaceae	Exosomes	HDFs, HUVECs, human keratinocytes	30–200 μg/ml	Promoted cell proliferation and migration at 30–200 μg/ml. Increased tube formation in HUVECs and promoted collagen synthesis in HDFs at 200 μg/ml	Sahin et al. (2019)
		A small peptide (YDWPGGRN)	Rats with full thickness wound	Topically applied 20 μL peptide (250 μM)	Stimulated angiogenesis and collagen production in wounds	Sui et al. (2020)

### Angelicae Dahuricae Radix

Angelicae dahuricae radix, the dried root of *Angelica dahurica* (Hoffm.) Benth. & Hook. f. ex Franch. & Sav [Apiaceae] or *Angelica dahurica* var. *formosana* (H.Boissieu) Yen [Apiaceae], is a widely used traditional Chinese medicine, which had been reported to possess wound healing activities. In the study conducted by Zhang *et al.*, Angelicae dahuricae radix 70% ethanolic extract (1.2 g/kg once daily by oral gavage) was proven to be able to accelerate diabetic wound healing *via* inducing angiogenesis (Zhang XN. et al., 2017). Another study conducted in db/db mice with full-thickness cutaneous wound found that Angelicae dahuricae radix 70% ethanolic extract treatment (1.8 g/kg once daily by oral gavage) resulted in the reduced wound area, increased neovascularization, elevated PDGF-β expression, and increased capillary formation (Guo et al., 2020). Its potential mechanisms were studied using human umbilical vein endothelial cells (HUVECs) and results showed that Angelicae dahuricae radix extract stimulated the angiogenesis *via* the activation of ERK1/2, PI3K/Akt, and eNOS/NO signaling pathways (Zhang XN. et al., 2017) as well as the HIF-1α/PDGF-β signaling pathway (Guo et al., 2020).

### Angelicae Sinensis Radix

Angelicae sinensis radix, the dried root of *Angelica sinensis* (Oliv.) Diels [Apiaceae], is a famous botanical drug, which has been used for a long time in East Asia. Nowadays, Angelicae sinensis radix is also used as a functional food in Europe and America. Promoting angiogenesis is one of the biological activities of Angelicae sinensis radix (Majewska and Gendaszewska-Darmach, 2011). Thus, increasing attention is drawn on the wound healing activities of Angelicae sinensis radix. In the research conducted by Lam et al. the angiogenic activities of Angelicae sinensis radix extract (containing 60% polysaccharide) were studied in both HUVECs and the zebrafish model (Lam et al., 2008). Angelicae sinensis radix extract at the dose of 11.1–100 μg/ml significantly stimulated the proliferation, migration, and tube formation of HUVECs *via* the activation of the JNK 1/2 and p38 signal pathways. In the zebrafish model, Angelicae sinensis radix extract (50–400 μg/ml) treatment stimulated angiogenesis in the subintestinal vessels. In Hsiao’s research, the proteomic approach was applied to elucidate the wound healing mechanism of Angelicae sinensis radix (Hsiao et al., 2012a). Fifty-one differentially expressed protein spots were observed after human embryonic skin fibroblasts were treated with Angelicae sinensis radix extract (300 μg/ml). Among all the differentially expressed proteins, peroxiredoxins (PRDX-2, PRDX-4, and PRDX-6), Parkinson’s disease protein 7 (PARK7), and glutathione S-transferase Pi (GSTP1) were the ones involved in antioxidant activity. Proteomic results showed that Angelicae sinensis radix exhibited antioxidative activity *via* regulating the expression of PRDX-2, PRDX-4, PRDX-6, PARK7, and GSTP1 (Hsiao et al., 2012a). Additionally, Angelicae sinensis radix upregulated HSPB1 to inhibit cell apoptosis and regulate cell mobility; upregulated annexin A2 and VAT-1 to regulate calcium ion; downregulated NDKB and MARE1 to enhance the migration of fibroblasts; upregulated CAPNS1 to promote cell growth; and upregulated glycolysis to provide extra energy for wound healing processes (Hsiao et al., 2012a).

SBD.4 was a low-molecular weight fraction prepared from Angelicae sinensis radix (Zhao et al., 2006). In both the genetically diabetic mouse wound model and the human skin/severe-combined immunodeficiency (SCID) mouse wound model, the topical application of SBD.4 (2 mg per wound) in 2% carboxymethyl cellulose showed excellent wound healing activities (compared with becaplermin). In another study conducted by Zhao’s team, wound healing activity of SBD.4 on tissue healing was evaluated under the form of a SBD.4-nanosilver hydrocolloid wound dressing (Zhao et al., 2012). Results showed that SBD.4 dressing (containing 1% SBD.4) can increase type I collagen production in human dermal fibroblasts (HDFs), induce angiogenesis in zebrafish, and promote the healing of patients with chronic lower extremity ulcers. To sum up, SBD.4 formulated in wound dressings may benefit the patients with chronic ulcers.

### Astragali Radix

Astragali radix, the dried root of *Astragalus mongholicus* Bunge [Fabaceae], is a traditional Chinese medicine used to enhance immune system function and promote wound healing (Han et al., 2009). Several components had been isolated from Astragali radix and studied for the wound healing activities including formononetin (Huh et al., 2011), astragaloside IV (Luo et al., 2016), and a polysaccharide named APS2-1 (Zhao et al., 2017).

Formononetin is a phytoestrogen isolated from Astragali radix. According to Huh’s research, formononetin (50 μM) was injected into dermis that is nearby wound, and it significantly promoted the wound healing in mouse full-thickness excisional wound (Huh et al., 2011). Mechanism studies revealed that growth factors including TGF-β1, VEGF, PDGF, and bFGF as well as Egr1 were significantly increased in HUVECs after formononetin treatment (0.1–50 μM) (Huh et al., 2011). Furthermore, western blot assays proved that the activation of Egr-1 by formononetin could be suppressed by ERK inhibitor and p38 inhibitor, suggesting that formononetin promoted wound healing by the regulation of Egr-1 *via* the ERK and p38 MAPK pathways (Huh et al., 2011).

Astragaloside IV (AS-IV) is a saponin derived from Astragali radix. Results of Luo’s study indicated that local delivery of AS-IV (100 mM) once daily promoted the wound closure in the diabetic mice skin wounds (Luo et al., 2016). On day 10 after wounds, wound closures in AS-IV-treated mice and vehicle treated mice were 72 ± 4.1% and 48.2 ± 3.1%, respectively. In-depth study found that AS-IV promoted wound healing in four parts: re-epithelialization, extracellular matrix deposition, neovascularization, and alternatively activated macrophages development. In the part of re-epithelialization, the AS-IV-treated group showed higher re-epithelialization rate (about 75%) than the untreated group (about 50%) (Luo et al., 2016). In the part of extracellular matrix deposition, the AS-IV-treated group increased the production of collagen compared to the untreated control group on Day 3 (Luo et al., 2016). In the part of neovascularization, more CD31 (a marker of angiogenesis) was detected in AS-IV-treated wounds (Luo et al., 2016). In the part of alternatively activated macrophages development, AS-IV-treated group increased the number of F4/80^+^CD206^+^ cells (an alternatively activated macrophages) and the expression of arginase-1 and Ym1 (Luo et al., 2016).

APS2-1 is a polysaccharide isolated from Astragali radix, which can promote wound healing *via* reducing the inflammatory response, promoting cell cycle progression, and the secretion of cytokines (Zhao et al., 2017). In the part of inflammatory, IκBα is a proinflammatory cytokine and APS2-1 (25 μg/ml) treatment significantly decreased the level of phosphorylation of IκBα in human skin fibroblast cells (Zhao et al., 2017). In the part of cell cycle progression, APS2-1 (1, 5 and 25 μg/ml) decreased the percentage of human skin fibroblast cells in the G0/G1 phase and increased the percentage of cells in the S and G2/M phases, leading to the promoted cell cycle progression significantly (Zhao et al., 2017). In the part of secretion of cytokines, the topical application of 0.5 g ointment (containing 1.55% APS2-1) increased the secretion of EGF, bFGF, and TGF-β1 in mice wounds (Zhao et al., 2017).

### Draconis Sanguis

According to Chinese Pharmacopoeia, Draconis sanguis is a red resin obtained from *Calamus draco* Willd. [Arecaceae]. In other cultures, red resins obtained from *Dracaena cochinchinensis* (Lour.) S.C.Chen [Asparagaceae], *Croton salutaris* Casar [Euphorbiaceae], *Pterocarpus officinalis* Jacq [Fabaceae] *etc.* were also considered as Draconis sanguis, and they have been widely used in traditional medicine since ancient times (Pona et al., 2019). According to *Compendium of Materia Medica*, Draconis sanguis showed wound healing activity *via* promoting tissue regeneration. *In vitro* and *in vivo* wound healing activities of Draconis sanguis had been proven by several studies, and Draconis sanguis exhibited wound healing activities *via* promoting angiogenesis, collagen deposition, epithelization, and wound contraction (Liu et al., 2013b; Apaza Ticona et al., 2020). Additionally, in a case report, a patient who suffered from chronic pressure ulcer with tunneling received an external application of Draconis sanguis powder (Ji et al., 2015). In addition to Draconis sanguis powder treatment, conventional treatments such as local oxygen therapy and anti-infection therapy were also used in this patient. The patient’s integrative treatment program led to complete amelioration of the pressure ulceration (Ji et al., 2015).

By bio-guided isolation, bexarotene, taspine, and 2-hydroxy-1-naphthaldehyde isonicotinoyl hydrazone were isolated from Draconis sanguis and considered to be the active constituents (Apaza Ticona et al., 2020). NF-κB inhibitory activities of these three constituents in THP-1 (human peripheral blood monocyte), HaCaT (human skin keratinocyte), and NIH-3T3 (mouse embryo fibroblast) cells were studied. Results showed that bexarotene, taspine, and 2-hydroxy-1-naphthaldehyde isonicotinoyl hydrazone exhibited NF-κB inhibitory activity in all these 3 cell lines with IC_50_ values of 0.10–4.78 μM (Celastrol as positive control with an IC_50_ of 7.96 μM). The agar well diffusion method was used to study the antimicrobial activities of these three constituents. Results showed that bexarotene, taspine, and 2-hydroxy-1-naphthaldehyde isonicotinoyl hydrazone showed higher antimicrobial activities than ofloxacin (positive drug) against *S. aureus*, *E. coli*, and *C. albicans* strains (Apaza Ticona et al., 2020). MIC values of bexarotene, taspine, 2-hydroxy-1-naphthaldehyde isonicotinoyl hydrazone, and ofloxacin were 0.12–0.16, 0.31–0.39, 3.96–3.99, and 27.56 μM, respectively. Meanwhile, bexarotene, taspine, and 2-hydroxy-1-naphthaldehyde isonicotinoyl hydrazone could also stimulate the proliferation of fibroblasts (NIH3T3 cells) and keratinocytes (HaCaT cells) (Apaza Ticona et al., 2020).

Dracorhodin perchlorate (DP), a synthetic analog of dracorhodin, which is isolated from Draconis sanguis, showed various pharmacological activities. Animal studies showed that DP was able to promote wound healing in rats *via* inhibiting the section of IL-1α and TNF-α, stimulating the expression of VEGF and TGF, and regulating fibroblast proliferation (Jiang et al., 2017; Jiang et al., 2018). *In vitro* studies demonstrated that DP (1 and 2 μg/ml) enhanced HaCaT keratinocytes wound healing *via* β-catenin, ERK/p38, and AKT signaling pathways (Lu et al., 2021). On human umbilical vein endothelial cells cultured under high glucose (25 mM) stimulation, DP (7.5 μM) exhibited angiogenic activity *via* the Ras/MAPK pathway (Li F. et al., 2016). Meanwhile, DP (0.5–4 μg/ml) could also stimulate fibroblast proliferation *via* the activation of EGFR and its downstream ERK/CREB and PI3K/AKT/mTOR pathways (Liu et al., 2019). Taken together, DP may be developed into a potential lead compound for the treatment of skin wounds.

### Notoginseng Radix et Rhizoma

Notoginseng Radix et Rhizoma [Araliaceae; *Panax notoginseng* (Burkill) F.H.Chen] has been used for hundreds of years in traumatic injuries and various kinds of bleeding (Ren et al., 2020). Notoginseng Radix et Rhizoma is an important component of Yunnan Baiyao, and Yunnan Baiyao is a famous commercial medicinal product in China, which is widely used for wounds (Yao et al., 2021). Saponins isolated from Notoginseng Radix et Rhizoma are the active components. Saponins could enhance angiogenesis on HUVECs *in vitro* and zebrafish *in vivo via* the activation of the VEGF-KDR/Flk-1 and PI3K-Akt-eNOS signaling pathways (Hong et al., 2009). Another mechanism of promoting angiogenesis activity is related to AMPK and eNOS-dependent pathways (Wang et al., 2017). Yu’s research found out that saponins can promote the proliferation of anterior cruciate ligament fibroblasts and increase the expression of collagen and fibronectin *via* enhancing the phosphorylation of PI3K, AKT, and ERK (Yu et al., 2015). Si-ye’s investigation showed saponins can inhibit scar formation through suppressing the proliferation of fibroblast *in vitro* and reducing the expression of α-SMA in a murine model of cutaneous wound (Men et al., 2020). Meanwhile, saponins can inhibit the formation of hypertrophic scar through inhibiting extracellular matrix deposition and stimulating cell apoptosis by modulating the PIK3/AKT signaling pathway (Zhi et al., 2021).

### Arnebiae Radix

Arnebiae radix is the dried root of *Arnebia euchroma* (Royle ex Benth.) I.M.Johnst [Boraginaceae], *Arnebia guttata* Bunge [Boraginaceae] or *Lithospermum erythrorhizon* Siebold & Zucc [Boraginaceae]. *Arnebia euchroma* (Royle ex Benth.) I.M.Johnst [Boraginaceae] mainly grows in Xinjiang of China. *Arnebia guttata* Bunge [Boraginaceae] mainly grows in inner Mongolia of China, and *Lithospermum erythrorhizon* Siebold & Zucc [Boraginaceae] grows widely in China, Korea, and Japan. Present research studies proved that Arnebiae radix and shikonin, an important component of it, possessed the activity of wound healing (Andujar et al., 2013).

In Hsiao’s research, a proteomic platform was applied to explore the wound healing activity of *Lithospermum erythrorhizon* Siebold & Zucc. (Hsiao et al., 2012b). Results showed that 95% ethanol extract of *Lithospermum erythrorhizon* Siebold & Zucc. (2.5–20 μg/ml) can promote cell viability, increase antioxidant capacity, and reduce cell mobility of fibroblasts. The molecular mechanism study revealed that *Lithospermum erythrorhizon* Siebold & Zucc., 95% ethanol extract can downregulate 10-MARE1 and 11-CLIC1 to inhibit cell mobility, upregulate 13-NME1 and p-p38 to promote cell proliferation, upregulate 20-PRDX4 to produce antioxidant activity, and upregulate 21PGK1 and downregulate 18-PSME1 to coordinate metabolism (Hsiao et al., 2012b).

In a study conducted by Karayannopoulou et al., the wound healing activity of an alkannins/shikonins (A/S)-based ointment was evaluated in dogs with surgically created full-thickness skin defects. A/S-based ointment was a pharmaceutical formulation, containing isohexenylnaphthazarins approved by Hellenic Health Authorities. In dog full-thickness cutaneous wounds, the topical application of A/S-based ointment significantly promoted angiogenesis, collagen production, and epithelialization in wounds, but the wound healing time was not shortened (Karayannopoulou et al., 2011). One of the shikonin’s mechanisms in wound healing was anti-inflammatory. Yan’s research showed that shikonin (1 μM) can inhibit the translocation of NF-κB from cytoplasm to nucleus induced by TNF-α in fibroblasts (Yan et al., 2015). Imai’s team focused on the pharmacologicial activities of shikonin in human gingival fibroblasts (Imai et al., 2019). Results showed shikonin (1 and 10 μM) can promote the growth and migration of human gingival fibroblasts. Meanwhile, shikonin (1 μM) increased the production of Type I collagen and the gene expression of VEGF and FN (a cell adhesion factor) in human gingival fibroblasts (Imai et al., 2019). By using the ERK1/2 inhibitor PD98059, the aforementioned activities of shikonin were reduced significantly, indicating that the mechanisms of the biological activities were associated with the activation of the ERK1/2 signaling pathway (Imai et al., 2019). Epithelial–mesenchymal transition (EMT) is another factor that contributes to wound healing (Lamouille et al., 2014). According to Yin’s research, shikonin upregulated the specific EMT regulatory molecules and downregulated the expression of microRNA-205 and other microRNAs in mice wounds, which indicated that shikonin can stimulate EMT and suppress the expression of the associated microRNAs in skin wound healing (Yin et al., 2013).

### Bletillae Rhizoma

Bletillae Rhizoma is the tuber of *Bletilla striata* (Thunb.) Rchb. f [Orchidaceae], which has been widely used in China for the treatment of hemoptysis, traumatic bleeding, ulcers, and chapped skin (He et al., 2017). Phytochemical studies revealed that polysaccharides were the major chemical constituents and Bletillae Rhizoma polysaccharides (BRP) not only promoted wound healing but also showed excellent performance as a promising natural biomaterial (He et al., 2017).

In Zhang’s research, the wound healing activity of BRP was investigated both *in vitro* and *in vivo* (Zhang C. et al., 2019). In *in vitro*, BRP (5 and 10 μg/ml) enhanced the proliferation and migration of mouse fibroblast cells. In *in vivo*, BRP hydrogel topically applied once daily at the dose of 0.4 g/kg significantly promoted the wound healing process in mouse full-thickness excision wound. In Luo’s study, BRP hydrogel was prepared by the oxidation and crosslinking method and 12.5%-crosslinked BRP hydrogel was applied on the mouse cutaneous wound bed (Luo et al., 2010). In the BRP hydrogel-treated wound tissues, the number of inflammatory cells and the level of TNF-α were significantly decreased and the re-epithelization was improved. *In vitro* mechanism studies showed that BRP at 80 μg/ml promoted the vascular endothelial cell proliferation and VEGF expression (Wang et al., 2006). In RAW264.7 cells, BRP (5–200 μg/ml) stimulated the expression of iNOS, TNF-α, and IL-1β in concentration-dependent manner (Diao et al., 2008). A recent study conducted in the diabetic mouse model found that 5% BRP solution (50 μl) treatment once daily could accelerate the diabetic wound healing *via* inhibiting the high glucose-activated NLRP3 inflammasome (Zhao et al., 2021). Additionally, BRP could also be used for the treatment of oral ulcer (Liao et al., 2019). Would dressings based on BRP such as chitosan-Ag nanoparticles and chitosan-BSP spongy bilayer dressing, BRP/carboxymethyl chitosan/Carbomer 940 hydrogel, and probiotic-bound oxidized BRP-chitosan composite hydrogel were all promising wound dressings which could be applied for wound healing (Ding et al., 2017; Huang Y. et al., 2019; Zhang Q. et al., 2019; Yang L. et al., 2020).

### Rhei Radix et Rhizoma

Rhei Radix et Rhizoma, the dried root and rhizome of *Rheum palmatum* L [Polygonaceae], *Rheum tanguticum* (Maxim. ex Regel) Balf [Polygonaceae], or *Rheum officinale* Baill [Polygonaceae], is a traditional botanical drug recorded in Chinese Pharmacopoeia, which is widely distributed in China and famous for its remarkable pharmacological activities, such as anti-inflammatory, antimicrobial, and hemostatic activities (Wang et al., 2012). Emodin is an anthraquinone derivative isolated from Rhei Radix et Rhizoma, which has been proven to possess wound healing activity. Wounds treated with topical emodin (100–400 μg/ml) showed higher content of hydroxyproline and more tensile strength (Tang et al., 2007). Further molecular mechanism study indicated that emodin could stimulate the tissue regeneration *via* regulating the Smads-mediated TGF-β_1_ signaling pathway (Tang et al., 2007).

In the studies conducted by Yang et al., Rhei Radix et Rhizoma and Angelicae dahuricae radix 70% ethanol extract were mixed together at the ratio of 1:1 as a mixture. The wound healing activity of the mixture was evaluated in rat excisional wound non-infected or infected with *Staphylococcus aureus* (Yang et al., 2017; Yang WT. et al., 2020). In non-infected wound, the topical application of the mixture significantly promoted the wound healing, and more collagen, myofibroblasts, and inflammatory cell infiltration were observed in the wound site tissues treated with the mixture. Increased plasma IL-6 level and decreased TGF-β1 level were also observed in the group treated with the mixture (Yang et al., 2017). *In vitro* disc diffusion test results showed that 11.02 μg/disc of mixture solid had an average 8.13 ± 0.05 mm inhibition zone against *Staphylococcus aureus* ATCC 29213 (Yang WT. et al., 2020). In *Staphylococcus aureus*-infected wound, the mixture also exhibited excellent wound healing activity (Yang WT. et al., 2020). Taken together, the mixture of Rhei Radix et Rhizoma extract and Angelicae dahuricae radix extract could accelerate the bacterial-infected wound healing.

### Rehmanniae Radix

Rehmanniae radix is the root tuber of *Rehmannia glutinosa* (Gaertn.) DC [Orobanchaceae], which is widely distributed in China. According to *Compendium of Materia Medica*, Rehmanniae radix is recommended to treat acute mastitis. Nowadays, Rehmanniae radix has been widely applied in traditional Chinese medicine prescriptions for the treatment of wound healing. In Lau’s research, the wound healing activity of Rehmanniae radix was evaluated with a diabetic foot ulcer rat model (Lau et al., 2009b). By comparing H&E staining photographs of wound tissues from rats treated with water or Rehmanniae radix extract (1.85 g/kg), the granulation tissue treated with Rehmanniae radix extract was more solid than that treated with water, which implied that Rehmanniae radix extract can facilitate tissue regeneration. Meanwhile, Rehmanniae radix extract increased the production of VEGF, indicating RR can enhance angiogenesis (Lau et al., 2009b).

In order to further investigate the angiogenesis activity of Rehmanniae radix, a zebrafish model was applied (Liu et al., 2011; Liu et al., 2014). Several Rehmanniae radix fractions were obtained and sub-fraction C2 (6.25–12.5 μg/ml) showed the most potent angiogenesis activity (Liu et al., 2011). Then, sub-fraction C2 was proceeded for further isolation and the major compound was identified as norviburtinal. In the zebrafish model, norviburtinal showed significant angiogenesis activity at the concentration of 50 μg/ml (Liu et al., 2011). Acteoside is another active component isolated from Rehmanniae radix, which exhibits wound healing activity (Nan et al., 2018). After normal human dermal fibroblasts were treated with acteoside (6.3–100 µM), proMMP-2 was activated along with an increase in MT1-MMP expression *via* the PI3K signal pathway.

### Salviae Miltiorrhizae Radix et Rhizoma

Salviae Miltiorrhizae Radix et Rhizoma, a traditional Chinese botanical drug, is the root and rhizome of *Salvia miltiorrhiza* Bunge [Lamiaceae], which has been widely applied for the treatment of various diseases, including traumatic injuries, cardiovascular, and cerebrovascular diseases. In Irmak’s research, the rat burn model was applied to investigate the wound healing activity of Salviae Miltiorrhizae Radix et Rhizoma (Irmak et al., 2018). Nonalcoholic Salviae Miltiorrhizae Radix et Rhizoma solution was orally administered at the dose of 1 g/kg/day for 14 days, and the solution was commercially produced with a 1:3 dry herb/menstruum ratio. Results showed that the average of the neovascularization score of the Salviae Miltiorrhizae Radix et Rhizoma solution treated group (2 ± 0.66) was significantly increased when compared with the control group (1.4 ± 0.51). Meanwhile, an increase in the tissue perfusion was observed in the Salviae Miltiorrhizae Radix et Rhizoma solution treated group. Numerous reports have demonstrated that inadequate tissue perfusion exacerbated the tendency toward burn wound deepening (Singh et al., 2007). The aforementioned results suggested that Salviae Miltiorrhizae Radix et Rhizoma can decrease the amount of necrosis in burn wounds and promote wound healing.

Cryptotanshinone (CT) is a terpenoid isolated from Salviae Miltiorrhizae Radix et Rhizoma. Min’s team designed a study using the excisional wound splinting model in db/db mice to evaluate the wound healing activity of CT (Song et al., 2020). Results showed that 300 mg/kg/d CT by gavage for 16 days can significantly accelerate the rate of wound closure, which displayed in the increase of re-epithelialization and granulation tissue formation. Mechanism studies showed that in diabetic mice, CT can depress leukocyte infiltration, decrease the expression of chemokine, increase eNOS phosphorylation, increase the protein expression of VEGF, Ang-1, inhibit MMP2 and MMP9 protein expression, and increase fibroblasts translation, leading to improved angiogenesis and collagen deposition (Song et al., 2020). Meanwhile, CT could also prevent scarring *via* decreasing the excessive deposition of extracellular matrix components (Li Y. et al., 2016). In addition to CT, Danshensu (3,4-dihydroxyphenyllactic acid) and salvianolic acid B were also proven to be active components in Salviae Miltiorrhizae Radix et Rhizoma for wound healing (Chen et al., 2014). In Detroit 551 human normal fibroblast cells, danshensu (25–200 µM) or salvianolic acid B (25–200 µM) treatment significantly promoted the cell proliferation. Danshensu (0.1 mM) or salvianolic acid B (0.1 mM) treatment increased the collagen production in fibroblasts. These results indicated that danshensu and salvianolic acid B could be utilized as wound healing agents.

### Lilii Bulbus

Lilii Bulbus, the dried fleshy petal-like layers of *Lilium lancifolium* Thunb [Liliaceae], *Lilium brownii* var. *viridulum* Baker [Liliaceae], or *Lilium pumilum* Redouté [Liliaceae], is a famous botanical drug widely used in China. Lilii Bulbus possesses anti-inflammatory and antibacterial activities, which are useful in wound repair (Zhou et al., 2021). In Esposito’s research, two steroidal glycosides from the Lilii Bulbus can accelerate wound closure in the 3T3 murine fibroblast cell line. Steroidal glycoside 1 was identified as (22R, 25R)-spirosol-5-en-3β-yl O-α-L-rhamnopyranosyl-(1→2)-β-D-glucopyranosyl-(1→4)-β-D-glucopyranoside and steroidal glycoside 2 was an acetylated derivative of steroidal glycoside 1, which was identified as (22R, 25R)-spirosol-5-en-3β-yl O-α-L-rhamnopyranosyl-(1→2)-[6-O-acetyl-β-D-glucopyranosyl-(1→4)]-β-D-glucopyranoside. Steroidal glycoside 1 accelerated the scratch wound closure in skin fibroblasts at the concentration of 0.2, 1, and 5 μM, and steroidal glycoside 2 showed wound healing activity at a concentration of 5 μM. The mechanism study showed that these two steroidal glycosides (5 μM) could induce the production of NO and increase the mRNA level of TGF-β Type I receptor, which played important roles in early wound healing (Esposito et al., 2013). The wound healing activity of steroidal glycoside 1 was further studied (Di et al., 2020). After the wounded fibroblast cells were treated with steroidal glycoside 1 at 5 μM, genes related to inflammation, chemokine, and tissue remodeling were downregulated. The decreases in the expression of key genes contributed to an early resolution of the inflammation and shorten the early phase of wound healing (Di et al., 2020). Also, steroidal glycoside 1 upregulated the extracellular matrix and cell adhesion-related genes to regulate basic functions of cells in wound healing (Di et al., 2020).

### Glycyrrhizae Radix et Rhizoma

Glycyrrhizae Radix et Rhizoma is the dried root and rhizome of *Glycyrrhiza uralensis* Fisch. ex DC [Fabaceae], *Glycyrrhiza inflata* Batalin [Fabaceae] or *Glycyrrhiza glabra* L [Fabaceae]. It is the most common botanical drug in China, which can be used to treat hepatitis, cough, gastric ulcer, and wound (Kotian et al., 2018). It is also widely used in cosmetics and food ingredients and the major components of it are triterpene saponins and flavonoids (Li et al., 2020). Hao’s research showed that in the rat wound model, the topical application of Glycyrrhizae Radix et Rhizoma soluble polysaccharide collagen sponge (containing 72 μg polysaccharide) could increase the content of hydroxyproline in wounds, promote the proliferation of capillaries and fibroblasts in granulation tissues, and increase the number of microvessels in wound sites through activating the expression of p-STAT3 and VEGF and upregulating the transcription levels of VEGF mRNA and miRNA-21 genes (Hao et al., 2020). Another active component is isoliquiritin, an isoflavonoid isolated from Glycyrrhizae Radix et Rhizoma. According to Liu’s investigation, isoliquiritin at 100 or 200 μg/ml can accelerate the healing of zebrafish skin wound *via* promoting inflammation response and facilitating angiogenesis (Liu et al., 2020).

### Zingiberis Rhizoma Recens

Zingiberis Rhizoma Recens is the fresh rhizoma of *Zingiber officinale* Roscoe [Zingiberaceae], which is widely cultivated in China. According to *Compendium of Materia Medica*, Zingiberis Rhizoma Recens can promote the generation of new tissues. Plenty of studies revealed that gingerols, essential oils, and diarylheptanoids were the major components of Zingiberis Rhizoma Recens and Zingiberis Rhizoma Recens showed excellent antioxidant, antibacterial, antitumor, and antiinflammatory activities (Li et al., 2021). In the acetic acid-induced gastric ulcer rat model, orally administration of Zingiberis Rhizoma Recens aqueous extract (1.25, 2.5, and 5 g/kg) reduced the gastric ulcer area in dose-dependent manner *via* inhibiting the expression of the chemokines and TNF-α (Ko and Leung, 2010). *In vitro*, 10-shogaol from Zingiberis Rhizoma Recens promoted the proliferation of human normal epidermal keratinocytes and dermal fibroblasts and 2 μM of 10-shogaol showed the highest increase in the cells viability. In both keratinocytes and fibroblasts, 10-shogaol (2 and 10 μM) significantly enhanced the production of TGF-β, PDGF-αβ, and VEGF (Chen CY. et al., 2012). 6-dehydrogingerdione, another component of Zingiberis Rhizoma Recens also can upregulate the growth factor production, and accelerate cellular proliferation and migration at 2 or 10 μM. The mechanism study revealed that 6-dehydrogingerdione could block the MAPK pathway by suppressing c-Jun protein level and ERK phosphorylation in fibroblasts (Chen CY. et al., 2013).

### Lonicerae Japonicae Flos

Lonicerae Japonicae Flos is the dried flower bud or opening flower of *Lonicera japonica* Thunb [Caprifoliaceae]. According to *Shen Nong’s Herbal Classic*, it was used in clinical applications for clearing heat and detoxification and it could also be used for the treatment of swelling and ulcer on the body surface. Modern research studies showed that Lonicerae Japonicae Flos possesses anti-inflammatory, antivirus and antioxidant activities, and organic acids, iridoid glycosides, flavonoids, and saponins were the main active compounds (Li J. et al., 2019). Wound healing activity of Lonicerae Japonicae Flos in the rat excision wound model was investigated by Chen WC. et al. (2012), and 10% (w/w) Lonicerae Japonicae Flos extract ointment was prepared by incorporating 10 g Lonicerae Japonicae Flos extract (90% ethanol) into 100 g of ointment base. Results showed that topical administration of 10% Lonicerae Japonicae Flos extract ointment significantly reduced the wound size, promoted the tissue regeneration, enhanced angiogenesis, and increased collagen deposition. Meanwhile, the elevated production of anti-inflammatory cytokine (IL-10) and suppressed production of proinflammatory cytokines (TNF-α and IL-6) were observed in rat blood samples after treated with Lonicerae Japonicae Flos extract ointment (Chen WC. et al., 2012). Since chlorogenic acid was the main active component of Lonicerae Japonicae Flos, another study conducted by Chen’s team investigated the wound healing activity of chlorogenic acid. In rats with excision wounds, topical application of 1% (w/w) chlorogenic acid ointment accelerated the wound healing through the upregulation of TNF-α and TGF-β (Chen WC. et al., 2013). Another study revealed that intraperitoneal administration of chlorogenic acid (25–200 mg/kg) could also accelerate wound healing (Bagdas et al., 2014).

### Portulacae Herba

Portulacae Herba is the dried aerial part of *Portulaca oleracea* L [Portulacaceae], which is widely distributed in the tropical and subtropical areas of the world. According to *Tang Materia Medica*, Portulacae Herba could be used for wounds. Modern pharmacological research studies demonstrated that Portulacae Herba possesses anti-inflammatory, antimicrobial, antioxidant, and antiulcerogenic activities (Zhou J. et al., 2015). Rashed’s team evaluated the preliminary wound healing activity of Portulacae Herba with the mouse excision wound model. Fresh Portulacae Herba homogenate (single dose of 50 mg or a twice dose of 25 mg topically applied) can stimulate wound contraction and increase the breaking strength of the treated wounds (Rashed et al., 2003). Additionally, a clinical study was conducted in lactating women to study the effects of *Portulaca oleracea* L. cream on the healing of nipple fissure (Niazi et al., 2019). The mean score of breast fissures significantly decreased and no complications were observed after the patients were treated with *Portulaca oleracea* L. cream (2%). It is worth noting that adverse events such as itching and tingling of whole body, dyspnea and tachycardia were reported after the systemic administration of *Portulaca oleracea* seed extract (Mirabzadeh et al., 2013).

### *Hippophae rhamnoides* L

*Hippophae rhamnoides* L [Elaeagnaceae] is a shrub native to various countries, including China, India, Nepal, and Russia*. Hippophae rhamnoides* L. has been widely used for the treatment of various diseases, such as cough, skin diseases, and inflammation (Pundir et al., 2021). It has been reported that the leaves, fruits, and seeds of *Hippophae rhamnoides* L. all possess wound healing activities (Gupta et al., 2005; Gupta et al., 2006; Upadhyay et al., 2009; Ito et al., 2014).

In rat full-thickness wound models, *Hippophae rhamnoides* L. leave extract significantly promoted the wound healing (Gupta et al., 2005; Kim and Lee, 2017). Mechanism studies conducted in rat burn wounds found that it showed positive pharmacological effects on different phases of the wound healing process (Upadhyay et al., 2011). After the burn wounds were treated with *Hippophae rhamnoides* L. leave extract (5% prepared in petroleum jelly) twice daily for 7 days, no evidence of wound bleeding, exudates, pus, or inflammation were observed at any time (Upadhyay et al., 2011). In the rat burn wound model, *Hippophae rhamnoides* L. leave extract could promote the angiogenesis *via* upregulating the expression of VEGF in regenerated tissue, stimulate the collagen synthesis, and increase the levels of antioxidants such as glutathione, superoxide dismutase, catalase, glutathione-S-transferase, and vitamin C in wounds (Upadhyay et al., 2011). Meanwhile, the *Hippophae rhamnoides* L. fruit pulp flavone and the seed oil also showed excellent wound healing activities (Gupta et al., 2006; Upadhyay et al., 2009; Ito et al., 2014).

### *Urtica dioica* L

*Urtica dioica* L [Urticaceae] is a botanical drug widely used for the treatment of various diseases, such as diabetes, hypertension, cardiovascular diseases, and prostate cancer (El Haouari and Rosado, 2019). Recently, *Urtica dioica* L. has been proven to possess wound healing activity. In Razika’s study, crude saponins were extracted from the leaves of *Urtica dioica* L. and topical application of the extract (20% in Vaseline) significantly promoted the wound healing process of rat excision wounds (Razika et al., 2017). The antioxidant activity of the crude saponins extract was also evaluated by *in vitro* diphenyl-picryl-hydrazyl test (DPPH) and excellent antioxidant activity (IC_50_ = 0.159 mg/ml), which was similar to that of ascorbic acid (*p* > 0.05) was observed (Razika et al., 2017). In another study conducted by Zouari Bouassida et al*.*, hydroethanolic extract of *Urtica dioica* L. was proven to show hemostatic and wound healing activities (Zouari Bouassida et al., 2017). Lupeol was identified as an active component in the extract, and lupeol had been reported to be able to improve re-epithelization during the wound healing process (Ammar et al., 2015). Thus, it was suggested that *Urtica dioica* L. showed wound healing activity, and lupeol was the potential therapeutic material basis.

### *Periploca forrestii* Schltr

*Periploca forrestii* Schltr [Apocynaceae] is a Chinese folk medicine, which has been historically used for the treatment of traumatic injuries (Huang M. et al., 2019). Recently, the molecular mechanism of its wound healing activities and the active components of it were revealed by the Liu’s group (Chen et al., 2018; Li Y. et al., 2019; Chen et al., 2019). *Periploca forrestii* Schltr. extract was separated by a macroporous resin column, and 65% ethanol eluted fraction (EPFE65) was tested for the wound healing activity. In their *in vitro* study, EPFE65 (50 μg/ml) significantly promoted the proliferation and migration of mouse fibroblast and stimulated the collagen synthesis in mouse fibroblast. In the *in vivo* study, topical application of EPFE65 hydrogel (containing 0.1% EPFE65) exhibited wound healing activity *via* enhancing the re-epithelialization and promoting the formation of complete dermis (Li J. et al., 2019). Further studies demonstrated that the regulation of Src mediated Mek/Erk and PI3K/Akt signaling pathways was the potential mechanism of the wound healing, and cardiac glycosides were the potential active components (Li Y. et al., 2019). Periplocin, the cardiotonic steroide isolated from *Periploca forrestii* Schltr., exhibited wound healing activity *via* the activation of Src/Erk and PI3K/Akt pathways both *in vitro* and *in vivo* (Chen et al., 2019).

### *Streptocaulon juventas* (Lour.) Merr

*Streptocaulon juventas* (Lour.) Merr [Apocynaceae] is a widely used folk medicine of the Dai minority in China and it is famous for its anti-inflammatory, anticancer, and wound healing activities (Anaya-Eugenio et al., 2019). In the research conducted by the Nguyen’s group, the wound healing activity of *Streptocaulon juventas* (Lour.) Merr. root ethanolic extract was evaluated in the mouse excision wound model (Nguyen et al., 2017). Results showed that topical administration of the extract (100 mg/kg/day) remarkably reduced the wound closure time. Meanwhile, the reduced expression of TNF-α and NF-κB1 genes and enhanced angiogenesis were observed in the wound granulation tissues treated with *Streptocaulon juventas* (Lour.) Merr. extract (Nguyen et al., 2017). Taken together, it was deduced that *Streptocaulon juventas* (Lour.) Merr. extract exerted wound healing activity *via* inducing the fibroblast proliferation and angiogenesis, and inhibiting the inflammation.

### *Carthamus tinctorius* L

*Carthamus tinctorius* L [Asteraceae] is a traditional botanical drug for treating blood stasis and painful menstrual problems (Zhang LL. et al., 2016). The flower and seed are the main medicinal parts of *Carthamus tinctorius* L. Flavonoids, alkaloids, and organic acids are the components responsible for most of its pharmacological activities and hydroxysafflor yellow A (HSYA) is the most popular component (Zhang LL. et al., 2016). The wound healing activity of HSYA both *in vitro* and *in vivo* was evaluated by Gao et al. (2018). *In vitro*, HSYA (0.4, 0.8 and 1.6 mM) promoted the migration of human epithelial keratinocytes in a dose-dependent manner. Low concentration of HSYA (0.4 mM) increased the tube formation of HUVECs and high concentration of HSYA (1.6 mM) impaired the tube formation. In diabetic rats with splinted excisional wound, HSYA topically applied at the dose of 0.2 mg significantly promoted wound closure, re-epithelialization and angiogenesis. Meanwhile, the higher collagen content and elevated VEGF and TGF-β1 levels were observed in HSYA-treated wounds (Gao et al., 2018).

### *Reynoutria japonica* Houtt

*Reynoutria japonica* Houtt [Polygonaceae] is a popular botanical drug widely distributed in East China, Central South, and Southwest China. Since ancient time *Reynoutria japonica* Houtt. has been used for the treatment of jaundice, scald, inflammation, and favus (Peng et al., 2013). Izabela’s team did the research to evaluate the wound healing activity of *Reynoutria japonica* Houtt. by using human gingival fibroblasts (HGFs). The results showed the various extracts of *Reynoutria japonica* Houtt. (25% ethanol, 40% ethanol, or 60% acetone) all able to stimulate HGFs proliferation and migration, and increase collagen III synthesis at the dose of 50 μg/ml (Nawrot-Hadzik et al., 2021). Various compounds have been isolated from *Reynoutria japonica* Houtt, including quinones, stilbenes, flavonoids, coumarins, and other polyphenolic compounds (Peng et al., 2013). Resveratrol is one of the active components of *Reynoutria japonica* Houtt, which is used widely in skin care products to improve the overall condition of the skin (Igielska-Kalwat et al., 2019). Nowadays, various investigations have been conducted to demonstrate the mechanism for resveratrol’s wound healing activity. It is believed that resveratrol promotes the wound healing *via* attenuating the oxidative stress levels and increasing cell proliferation and migration (Kamolz et al., 2020).

### *Artemisia annua* L

In line with *Compendium of Material Medica*, *Artemisia annua* L [Asteraceae] could be used for wounds. *Artemisia annua* L. has been proven to possess antibacterial, antifungal, anti-inflammatory, and antioxidant activities by various studies (Ćavar et al., 2012). Owning to these properties, *Artemisia annua* L. has been used in novel wound dressings. In the study conducted by Mirbehbahani et al*.*, *Artemisia annua* L.-containing nanofibers were prepared, and the nanofibers promoted wound healing *via* encouraging the attachment, spreading and proliferation of the fibroblasts and exhibited antibacterial properties against *Staphylococcus aureus* (Mirbehbahani et al., 2020).

### *Acorus calamus* L

*Acorus calamus* L [Acoraceae] is a medicinal plant widely distributed in China. As recorded in *Compendium of Materia Medica*, in ancient times, *Acorus calamus* L. was a botanical drug used for traumatic injury. In traditional folk medicine of China and Indonesia, it was used for the treatment of inflammation, depression, hemorrhoids, skin diseases *etc*., and phenylpropanoids, sesquiterpenoids, and monoterpenes were the major constituents (Sharma et al., 2020). In rat excision and incision wounds, 80% ethanol extract of *Acorus calamus* L. dried leaves (20% or 40% ointment topically applied once daily) promoted wound healing *via* enhancing wound contraction, decreasing epithelialisation time, and increasing hydroxyproline content (Jain et al., 2010). The rhizoma of *Acorus calamus* L. also exhibited wound healing activity. Ponrasu’s team proved that after the dermal wounds in rat were treated with 70% ethanolic extract of *Acorus calamus* L. rhizoma (40 mg/kg topically applied once daily), the tensile strength of wounds was increased and granulation tissue was formed with more collagen, hexosamine, and uronic acid (Ponrasu et al., 2014).

### *Triticum aestivum* L

According to *Compendium of Material Medica*, *Triticum aestivum* L [Poaceae] (Wheat) had been used for wound healing since ancient time. Sahin’s team took the research in the wheat exosomes, and the wound healing activities of wheat grass juice-derived exosomes were evaluated *in vitro*. Results revealed that wheat exosomes (30–200 μg/ml) showed astonishing proliferative and migratory activities on HDFs, HUVECs, and human keratinocytes. Wheat exosomes significantly increased the expression level of collagen type I in HDFs and promoted the formation of tube-like structure of in HUVECs at the dose of 200 μg/ml (Sahin et al., 2019). Another interesting fraction of wheat is a small peptide named YDWPGGRN, which can accelerate wound healing in a rat model with a full thickness dermal wound through stimulating angiogenesis and collagen production in wounds (Sui et al., 2020).

## Multiherbal Preparations With Wound Healing Properties

It is believed that multiple botanical drug compositions in a particular ratio will reduce toxicity and give a better therapeutic effect. Thus, multiherbal preparations are widely used in Chinese hospitals. Many of the multiherbal preparations have been proven for their wound healing activities, such as Shiunko (Zi Yungao in Chinese) and NF3. Shiunko is a formulation consists of Arnebiae radix and Angelicae sinensis radix, which is developed during the Ming dynasty in China and widely used in China and Japan for the treatment of wounds (Chak et al., 2013). Mechanism studies revealed that Shiunko promoted the epithelization, angiogenesis, and granulation tissue formation of wounds (Huang et al., 2004; Lu et al., 2008; Chak et al., 2013). Recently, clinical trials proved that Shiunko might be also effective for radiation-induced dermatitis in patients with breast cancer and localized cutaneous leishmaniasis (Na-Bangchang et al., 2016; Kim et al., 2021). NF3, a two-botanical drug formulation comprised Astragali radix and Rehmanniae radix, is another one which has been extensively investigated for the treatment of diabetic foot ulcer (Lau et al., 2008; Lau et al., 2009a; Zhang et al., 2011; Lau et al., 2012). In the human skin fibroblast cell Hs27, NF3 (4 mg/ml) enhanced wound healing *via* activating the TGF-β pathway and promoting ECM deposition (Zhang et al., 2012). In human vascular endothelial cells, NF3 promoted cell migration at 75–300 μg/ml and enhanced tubule formation *via* MAPK and Akt pathways at 75 and 150 μg/ml (Liu et al., 2013a). A recent proof-of-concept study showed that NF3 treatment (5 g/sachet, two sachets daily) significantly reduced the foot ulcer area in Chinese patients with diabetes (Ko et al., 2014). Other multiherbal preparations with wound healing properties are summarized in Table 2.

**TABLE 2:** Multiherbal preparations and their wound healing activities.

Multiherbal preparation	Composition	Source or preparation procedure	Experimental model	Doses and route	Wound healing activity and potential mechanism	Reference
Jing Wan Hong ointment	30 drugs including Ampelopsis japonica (Thunb.) Makino [Vitaceae], Angelica dahurica (Hoffm.) Benth. and Hook.f. ex Franch. and Sav. [Apiaceae], *Lobelia* chinensis Lour. [Campanulaceae], Cinnamomum camphora (L.) J.Presl [Lauraceae], Atractylodes lancea (Thunb.) DC. [Asteraceae], Paeonia lactiflora Pall. [Paeoniaceae], and Conioselinum anthriscoides 'Chuanxiong’ [Apiaceae]	Commercially available and obtained from Tianjin Darentang Jingwanhong Pharmaceutical Co., Ltd	Nerve injury diabetic foot ulcer in rats	Topically applied with gauze immersed with the ointment	Promoted the foot ulcer healing through tissue regeneration, angiogenesis, and inflammation control, which was dependent on the increased expression of PDGF mRNA.	Jin et al. (2014)
Liu-He-Dan	More than 10 botanical drugs including but not limited to Rheum officinale Baill. [Polygonaceae], Phellodendron amurense Rupr. [Rutaceae], Angelica dahurica (Hoffm.) Benth. and Hook.f. ex Franch. and Sav. [Apiaceae], smoked plum, and Bletilla striata (Thunb.) Rchb.f. [Orchidaceae]	Drugs were powdered and mixed with water and honey into a paste	Patients with radiodermatitis associated with breast cancer	5 g Liu-He-Dan past applied externally once daily	Decreased inflammatory seepage and alleviated pain	Zhou et al. (2015b)
Tuo-Li-Xiao-Du-San	Comprised Angelica sinensis (Oliv.) Diels [Apiaceae], *Astragalus* mongholicus Bunge [Fabaceae], Angelica dahurica (Hoffm.) Benth. and Hook.f. ex Franch. and Sav. [Apiaceae], and thorns of Gleditsia sinensis Lam. [Fabaceae] in the ratio of 5:5:4:4	Crude botanical drugs were powered and then extracted by 70% ethanol for three times	Diabetic rats with full-thickness wound	Orally dosed once daily (1.5, 1.5, 1.2, and 1.2 g/kg for the four drugs)	Promoted diabetes-impaired wound healing *via* reducing inflammation, increasing angiogenesis and collagen deposition	Zhang et al. (2016c)
Ulcer oil	Comprised Phellodendron amurense Rupr. [Rutaceae] and Angelica dahurica (Hoffm.) Benth. and Hook.f. ex Franch. and Sav. [Apiaceae]	Obtained from the Beijing University of Chinese Medicine Dongzhimen Hospital	Diabetic rats with full-thickness wound	Topically applied (1 ml/cm^2^) once daily	Promoted wound healing *via* downregulating PTPIB and AGEs and upregulating VEGF and PDGF.	Jia et al. (2018)
Shengfu Oil	Comprised Phellodendron amurense Rupr. [Rutaceae], Rheum officinale Baill. [Polygonaceae], *Astragalus* mongholicus Bunge [Fabaceae], Coptis chinensis Franch. [Ranunculaceae], Scutellaria baicalensis Georgi [Lamiaceae], Angelica dahurica (Hoffm.) Benth. and Hook.f. ex Franch. and Sav. [Apiaceae], Boswellia sacra Flück. [Burseraceae], Sanguisorba officinalis L. [Rosaceae], Lithospermum erythrorhizon Siebold and Zucc. [Boraginaceae], Commiphora myrrha (T.Nees) Engl. [Burseraceae], Rehmannia glutinosa (Gaertn.) DC. [Orobanchaceae], Bletilla striata (Thunb.) Rchb.f. [Orchidaceae], Vincetoxicum atratum (Bunge) C.Morren and Decne. [Apocynaceae], Angelica sinensis (Oliv.) Diels [Apiaceae], and Cinnamomum camphora (L.) J.Presl [Lauraceae]	These botanical drugs were powdered and soaked in five times volume of sesame oil for 7 days at room temperature and then further incubated for another 2 h at 90°C. The filtered supernatant was Shengfu oil	Rabbit with full-thickness scalded wound	Topically applied at the dose of 0.15 ml/cm^2^ three times per day	Showed anti-inflammatory, analgesic, and antimicrobial activities. Promoted wound healing *via* regulating the expression of β-catenin, Dlk1, and COX-2	Chen et al. (2017)
Sheng-ji Hua-yu formula	Comprised eight drugs including *Astragalus* mongholicus Bunge [Fabaceae], Salvia miltiorrhiza Bunge [Lamiaceae], Rheum palmatum L. [Polygonaceae], *Calamus* draco Willd. [Arecaceae], Arnebia euchroma (Royle ex Benth.) I.M.Johnst. [Boraginaceae], Angelica dahurica (Hoffm.) Benth. and Hook.f. ex Franch. and Sav. [Apiaceae], and Nacre and Calamine. The amount of these drugs were 60, 15, 15, 10, 30, 30, 30, and 30 g, respectively	The ethanol extract of the drugs was prepared and then extracted with chloroform for four times. The extract was mixed, concentrated, and then mixed with carbomer	Diabetic mice with full-thickness wound	Topically applied the ointment at 0.5 g/cm^2^/day	Accelerated re-epithelialization and healing time of diabetic wounds *via* decreasing the high expression of activin/follistatin	(Kuai et al., 2018; Xiang et al., 2021)
Zhangpi Ointment	Comprised six botanical drugs and two minerals including *Glycyrrhiza* uralensis Fisch. ex DC. [Fabaceae], Angelica sinensis (Oliv.) Diels [Apiaceae], Arnebia euchroma (Royle ex Benth.) I.M.Johnst. [Boraginaceae], Rheum officinale Baill. [Polygonaceae], Rehmannia glutinosa (Gaertn.) DC. [Orobanchaceae], and Lycium barbarum L. [Solanaceae], rubber powder and calomel	Obtained from Shanghai Ninth People’s Hospital	Hydroxyurea-induced leg ulcers in patients with myeloproliferative neoplasms	About 1 mm thick ointment was applied to the surface of the ulcer	Compared with the control group, patients treated with Zhangpi Ointment achieved a significant higher rate of wound healing	Pang et al. (2018)
ANBP	Comprised Agrimonia eupatoria L. [Rosaceae], *Nelumbo nucifera* Gaertn. [Nelumbonaceae], Boswellia sacra Flück. [Burseraceae], and the pollen of *Typha* angustifolia L. [Typhaceae]	Powdered the drugs with ultralow temperature broken method	Rabbit ear hypertrophic scar model	Topically applied the powder	Promoted wound healing and reduced scar formation *via* the regulation of the TGF-b1/Smad pathway	Hou et al. (2014)
Huiyang Shengji decoction	Comprised *Astragalus* mongholicus Bunge [Fabaceae] 30 g, Atractylodes macrocephala Koidz. [Asteraceae] 10 g, Atractylodes lancea (Thunb.) DC. [Asteraceae] 10 g, Poria 10 g, Cinnamomum verum J.Presl [Lauraceae] 10 g, Aconitum carmichaeli Debeaux [Ranunculaceae] 10 g, Cervi Cornu Degelatinatum 10 g, Chaenomeles lagenaria (Loisel.) Koidz. [Rosaceae] 10 g, Sinapis alba L. [Brassicaceae] 10 g, Rehmannia glutinosa (Gaertn.) DC. [Orobanchaceae] 10 g, Angelica dahurica (Hoffm.) Benth. and Hook.f. ex Franch. and Sav. [Apiaceae] 10 g, and Glehnia littoralis (A.Gray) F.Schmidt ex Miq. [Apiaceae] 15 g	155 g drugs were soaked for 1 h and then extracted with water. The extract was filter and concentrated, and the concentration was 1.993 g/ml.Then the solution was freeze-dried into powder	db/db mice with full-thickness wound	Orally dosed of the decoction at 19.93 g/kg	Promoted the proliferation of epidermal cells in wounds *via* increasing EGFR expression and the activation of PI3K/AKT pathway	Liu et al. (2021)

## Discussion

**FIGURE 1:**
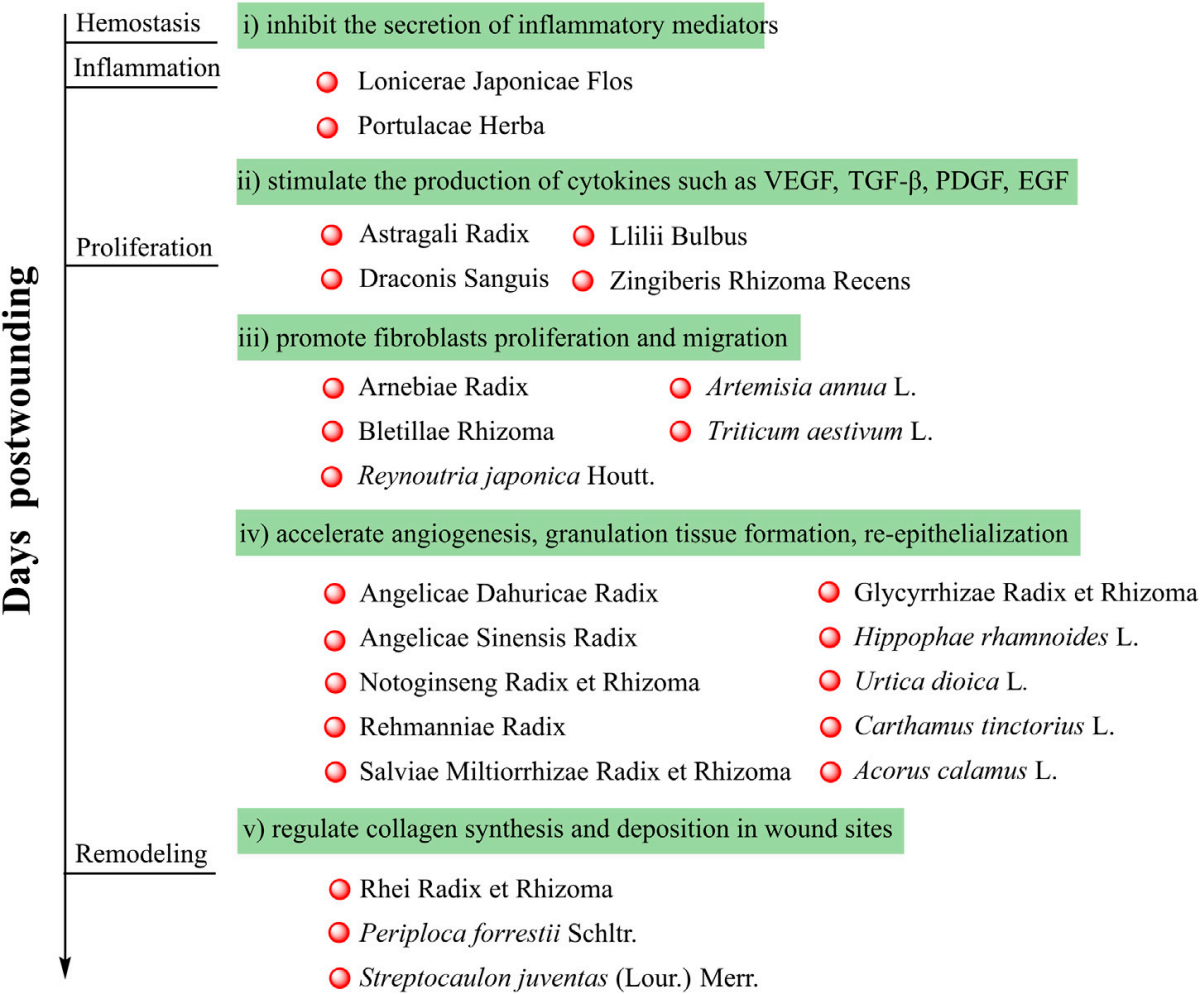
Botanical drugs in traditional Chinese medicine and their wound healing properties.

As described in this review, anti-inflammatory, antibacterial, antioxidant, proangiogenic, promoting fibroblast proliferation, and pro-collagen synthesis activities of botanical drugs contribute to the wound healing process. Botanical drugs in traditional Chinese medicine with these properties could i) inhibit the secretion of inflammatory mediators; ii) stimulate the production of cytokines such as VEGF, TGF-β, PDGF, and EGF; iii) promote fibroblasts proliferation and migration; iv) accelerate angiogenesis, granulation tissue formation, and re-epithelialization; and v) regulate collagen synthesis and deposition in wound sites, and all these lead to a promoted wound healing (Figure 1). Meanwhile, to maximize the therapeutic benefits of botanical drugs, different types of wound dressings include hydrogel, hydrocolloids, scaffolds, nanocomposites membranes, and nanofibers are used. Advanced wound dressings loaded with botanical drugs would be new treatment options.

**FIGURE 2:**
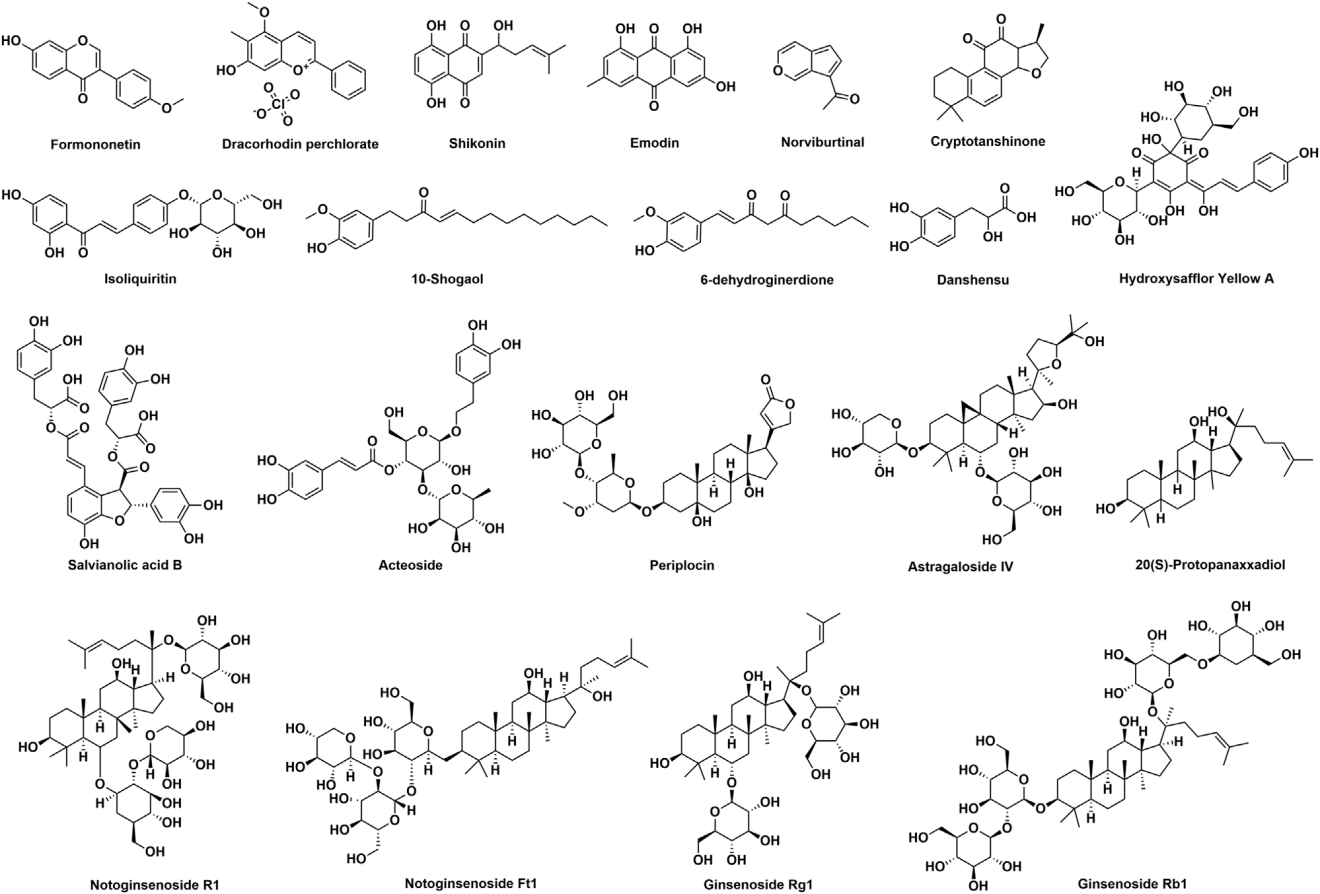
Chemical structures of components isolated from botanical drugs which possess wound healing activities.

Even though numerous investigations proved that botanical drugs are promising therapeutics for wound healing, there are still some limitations needed to be pointed out. First, for most of the botanical drugs, investigations about the wound healing activities remain at preclinical studies. Clinical trials are needed to prove the safety and effectiveness. There are many wound-related diseases in clinical, such as diabetic foot, venous leg ulcer, ulcerative bedsore, and burn ulcer*.* Well-designed clinical trials could provide scientific basis for further development and application of the botanical drugs for the treatment of wounds. Second, as for some botanical drugs, the molecular mechanisms are unclear. Along with the deepening of research, an increasing number of targets and pathways are demonstrated to be associated with wound healing. To better understand the wound healing activities of botanical drugs, further studies which are able to reveal the mechanism on the molecular biological levels are still needed. Third, extracts are commonly used to evaluate the wound healing activities of botanical drugs; however, components responsible for the activity are rarely identified (Figure 2), and the preparation of the extracts is usually not standardized. Due to the fact that botanical drugs obtained from different origins, habitats, and harvest time could be different in chemical profiles, it is important to identify the therapeutic basis or standardize the preparation of the extracts. Fourth, it is a general belief that botanical drugs display fewer side effects. Thus, most of the published investigations focused on the wound healing activities of botanical drugs, and the safety of using them for wounds was less discussed. Studies able to assess the potential adverse effects of botanical drugs for wounds are still needed. Taken together, botanical drugs are important sources of wound healing therapeutics and more efforts should be paid to bring these therapeutics earlier to patients.

## Conclusion

To sum up, botanical drugs in traditional Chinese medicine are powerful alternatives for the treatment of wounds. This review summarized the wound healing activities of traditionally used Chinese botanical drugs and multiherbal preparations. It provides valuable information for the development of effective wound healing drugs.
